# Supplementary figures and images for: Genotyping of familial Mediterranean fever gene (MEFV)—Single nucleotide polymorphism—Comparison of Nanopore with conventional Sanger sequencing
Source: PLoS One. 2022 Mar 17;17(3):e0265622. doi: 10.1371/journal.pone.0265622 (PMC8929590; doi:10.1371/journal.pone.0265622)

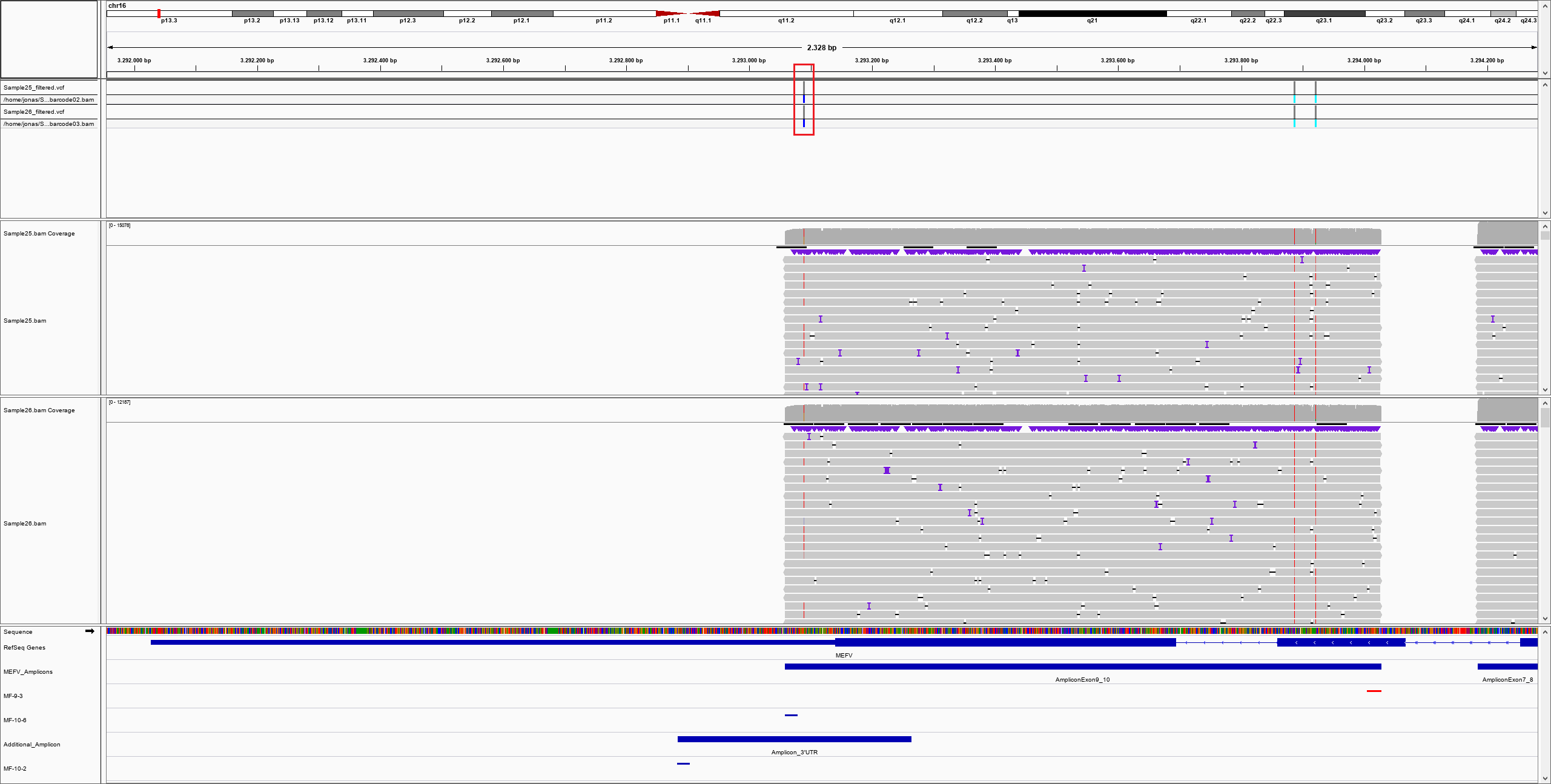

Supplement: S1 Fig — As Sanger sequencing shows a poor sequence quality at the start and end of a read, this region cannot be sequenced properly by using the routine Sanger sequencing workflow (MF-9-3 = forward sequencing primer Exon 9/10; MF-10-6 = reverse sequencing primer Exon 9/10). By sequencing an additional amplicon, which spans the region containing the variant, it was possible to confirm the transversion in both samples also by Sanger sequencing (MF-10-2 = forward sequencing primer 3’ UTR). (TIF) [file pone.0265622.s001.tif]

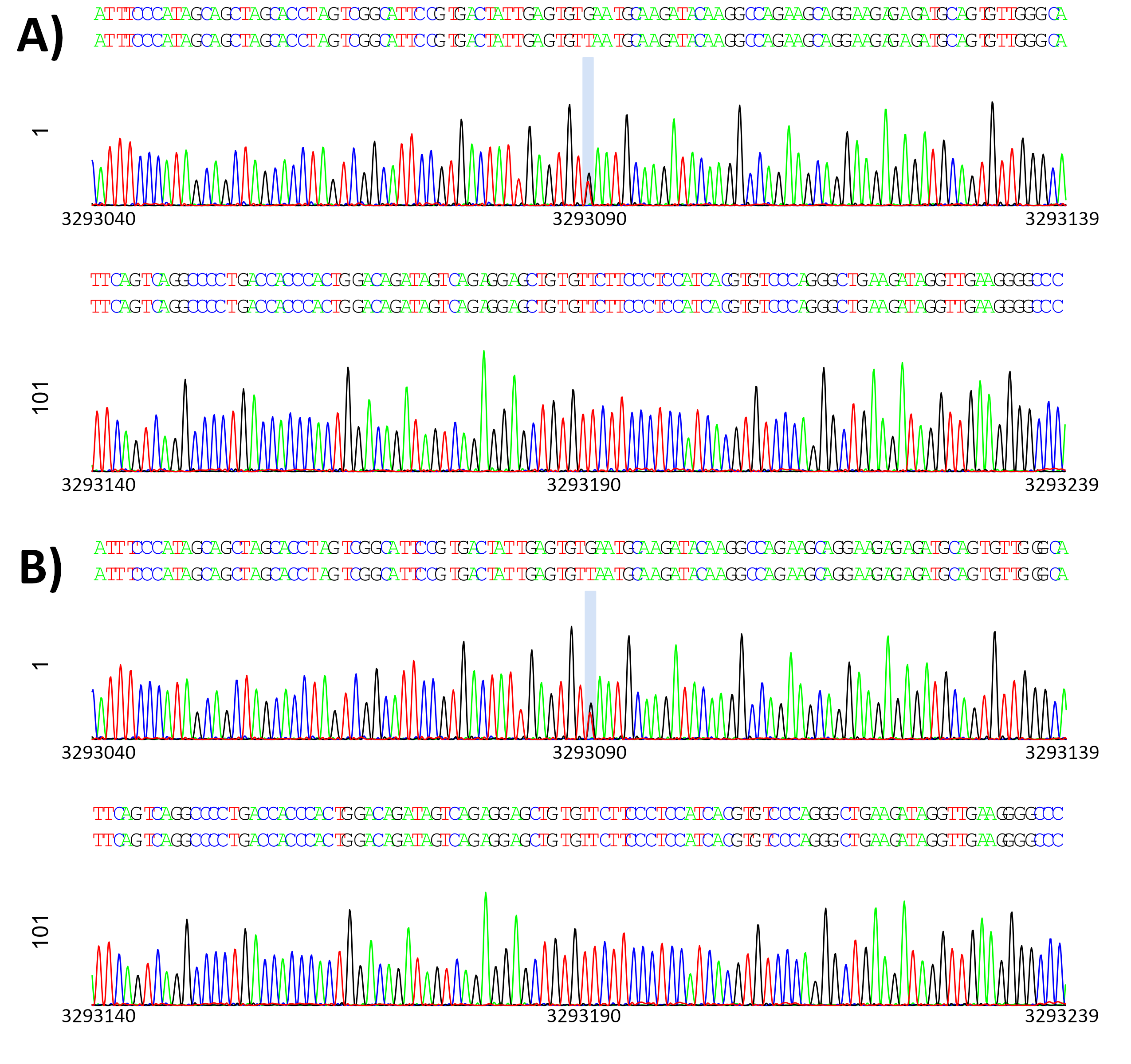

Supplement: S2 Fig — The transversion from guanine to thymine at genomic position 3293090 is clearly visible in sample 25 (A) and sample 26 (B). (TIFF) [file pone.0265622.s002.tiff]
