# Supplementary material for: Genotyping of familial Mediterranean fever gene (MEFV)—Single nucleotide polymorphism—Comparison of Nanopore with conventional Sanger sequencing
Source: PLoS One. 2022 Mar 17;17(3):e0265622. doi: 10.1371/journal.pone.0265622 (PMC8929590; doi:10.1371/journal.pone.0265622)
Supplement: S1 Table — (DOCX) [file pone.0265622.s003.docx]

| **Primer** | **Target** | **Sequence (5’ - 3’)** | **Primer length** | **Amplicon length** |
| --- | --- | --- | --- | --- |
| MF-1-5 | Exon 1 (forward) | CACATGTCTGCCAAGGCATG | 20 | 420 |
| MF-1-8 | Exon 1 (reverse) | TCAGAGTGAGCTGCTCTGAGCTC | 23 |  |
| MF-2-5 | Exon 2 (forward) | CTCTCCTCTGCCCTGAATCTTG | 22 | 846 |
| MF-2-6 | Exon 2 (reverse) | CAGGCTGGTCTCAAAGTCTTGG | 22 |  |
| MF-3-5 | Exon 3 (forward) | GGGCAGAGTCTAACTGAGAACTCG | 24 | 585 |
| MF-3-6 | Exon 3 (reverse) | CCAAGAATGCTGGTTAATGCACC | 23 |  |
| MF-4-5 | Exon 4 (forward) | CTTGCTACCAGAAGGAGATGTTCC | 24 | 343 |
| MF-4-8 | Exon 4 (reverse) | TTACCCTTGGCTGCTGGTTACC | 22 |  |
| MF-5-3 | Exon 5 (forward) | CCAGGAGGTGGGCTTCTG | 18 | 437 |
| MF-5-4 | Exon 5 (reverse) | CTATCCTAGGCCTTAGGGCTTCAC | 24 |  |
| MF-6-7 | Exon 6 (forward) | CCATTTCCCAGAAGGGATCAG | 21 | 458 |
| MF-6-8 | Exon 6 (reverse) | CTGACCAGATGCCCTTCTCCC | 21 |  |
| MF-7-1 | Exon 7/8 (forward) | TCATTTCCAGCTCACGGGTAC | 21 | 477 |
| MF-8-2 | Exon 7/8 (reverse) | CAAGTCAACAGCACAAGGGAAC | 22 |  |
| MF-9-3 | Exon 9/10 (forward) | GGGATTATACCCAACATAGCATGC | 24 | 970 |
| MF-10-6 | Exon 9/10 (reverse) | ACCTAGTCGGCATTCCGTGAC | 21 |  |
| MF-10-2 | 3’ UTR (forward)^a^ | CGGATTATGCAACGACTCCG | 20 | 381 |
| MF-10-3 | 3’ UTR (reverse)^a^ | TACATTCGCCAGCTGCTCTT | 20 |  |

^a^This target was only amplified in sample 25 and sample 26 to confirm a transversion which was initially only identified by Nanopore sequencing. UTR; untranslated region

**S1 Table. Specific primers used for the amplification of the targets within the MEFV gene.**
