# Supplementary material for: Genotyping of familial Mediterranean fever gene (MEFV)—Single nucleotide polymorphism—Comparison of Nanopore with conventional Sanger sequencing
Source: PLoS One. 2022 Mar 17;17(3):e0265622. doi: 10.1371/journal.pone.0265622 (PMC8929590; doi:10.1371/journal.pone.0265622)
Supplement: S2 Table — (DOCX) [file pone.0265622.s004.docx]

**S2 Table. PCR reaction mixes used for amplification of the targets within MEFV.**

Reaction mix for the amplification of exon 1, exon 4, exon 6, exon 7/8 or the 3’ UTR:

| **Reagent** | **Volume [µL]** |
| --- | --- |
| DNA template^a^ | 2.5 |
| AmpliTaq Gold 360 Master Mix | 12.5 |
| Forward primer (20 µM) | 0.5 |
| Reverse primer (20 µM) | 0.5 |
| Nuclease free water | 9.0 |
| **Total volume** | 25 |

^a^~60ng DNA template are used per reaction.

Reaction mix for the amplification of exon 2:

| **Reagent** | **Volume [µL]** |
| --- | --- |
| DNA template^a^ | 2.5 |
| AmpliTaq Gold 360 Master Mix | 12.5 |
| Q-Solution | 5.0 |
| Forward primer (20 µM) | 0.5 |
| Reverse primer (20 µM) | 0.5 |
| Nuclease free water | 4.0 |
| **Total volume** | 25 |

^a^~60ng DNA template are used per reaction.

Reaction mix for the amplification of exon 3, exon 5 or exon 9/10:

| **Reagent** | **Volume [µL]** |
| --- | --- |
| DNA template^a^ | 2.5 |
| AmpliTaq Gold 360 Master Mix | 12.5 |
| Enhancer | 2.5 |
| Forward primer (20 µM) | 0.5 |
| Reverse primer (20 µM) | 0.5 |
| Nuclease free water | 6.5 |
| **Total volume** | 25 |

^a^~60ng DNA template are used per reaction.
