# Supplementary material for: Genotyping of familial Mediterranean fever gene (MEFV)—Single nucleotide polymorphism—Comparison of Nanopore with conventional Sanger sequencing
Source: PLoS One. 2022 Mar 17;17(3):e0265622. doi: 10.1371/journal.pone.0265622 (PMC8929590; doi:10.1371/journal.pone.0265622)
Supplement: S3 Table — (DOCX) [file pone.0265622.s005.docx]

**S3 Table. PCR reaction programs used for the amplification of the targets within the MEFV gene.**

PCR reaction program used for the amplification of exon 1, exon 3, exon 4, exon 5, exon 6, exon 7/8, exon 9/10 and the 3’ UTR:

| **Step** | **Time [min]** | **Temperature [°C]** | **Cycle** |
| --- | --- | --- | --- |
| Initial Denaturation | 10:00 | 95 | - |
| Denaturation | 00:15 | 95 | 40 |
| Annealing | 00:20 | 62 |  |
| Extension | 00:30 | 72 |  |
| Final extension | 05:00 | 72 | - |

PCR reaction program used for the amplification of exon 2:

| **Step** | **Time [min]** | **Temperature [°C]** | **Cycle** |
| --- | --- | --- | --- |
| Initial Denaturation | 15:00 | 95 | - |
| Denaturation | 00:20 | 95 | 40 |
| Annealing | 00:30 | 62 |  |
| Extension | 00:30 | 72 |  |
| Final extension | 10:00 | 72 | - |
