# Supplementary material for: Genotyping of familial Mediterranean fever gene (MEFV)—Single nucleotide polymorphism—Comparison of Nanopore with conventional Sanger sequencing
Source: PLoS One. 2022 Mar 17;17(3):e0265622. doi: 10.1371/journal.pone.0265622 (PMC8929590; doi:10.1371/journal.pone.0265622)
Supplement: S4 Table — The final concentration in the reaction mix was 5 μM. (DOCX) [file pone.0265622.s006.docx]

| **Primer** | **Target** | **Sequence (5’ - 3’)** | **Length** |
| --- | --- | --- | --- |
| MF-1-5 | Amplicon exon 1 | CACATGTCTGCCAAGGCATG | 20 |
| MF-2-1 | Amplicon exon 2 | TGAGCAAACGCAGAGAGAAGG | 21 |
| MF-2-3 | Amplicon exon 2 | CTGCGCAGAAACGCCAG | 17 |
| MF-2-4 | Amplicon exon 2 | TTCGAAGGGCCTGCACTC | 18 |
| MF-3-6 | Amplicon exon 3 | CCAAGAATGCTGGTTAATGCACC | 23 |
| MF-4-5 | Amplicon exon 4 | CTTGCTACCAGAAGGAGATGTTCC | 24 |
| MF-5-3 | Amplicon exon 5 | CCAGGAGGTGGGCTTCTG | 18 |
| MF-6-7 | Amplicon exon 6 | CCATTTCCCAGAAGGGATCAG | 21 |
| MF-7-1 | Amplicon exon 7/8 | TCATTTCCAGCTCACGGGTAC | 21 |
| MF-9-3 | Amplicon exon 9/10 | GGGATTATACCCAACATAGCATGC | 24 |
| MF-10-6 | Amplicon exon 9/10 | ACCTAGTCGGCATTCCGTGAC | 21 |
| MF-10-2 | Amplicon 3’ UTR | CGGATTATGCAACGACTCCG | 20 |

**S4 Table. Sequencing primers which were used to sequence the individual amplicons by Sanger sequencing.** The final concentration in the reaction mix was 5 µM.
