# Supplementary material for: Genotyping of familial Mediterranean fever gene (MEFV)—Single nucleotide polymorphism—Comparison of Nanopore with conventional Sanger sequencing
Source: PLoS One. 2022 Mar 17;17(3):e0265622. doi: 10.1371/journal.pone.0265622 (PMC8929590; doi:10.1371/journal.pone.0265622)
Supplement: S5 Table — 47 samples were sequenced in four individual runs applying two R9.4.1 flow cells. (DOCX) [file pone.0265622.s007.docx]

**S5 Table: Overview of the barcode assignment which was used to sequence the clinical samples on a MinION sequencing device. 47 samples were sequenced in four individual runs applying two R9.4.1 flow cells.**

| **MinION run** | **Flow cell ID** | **Barcode^a^** | **Amplicon pool concentration [ng/µl]** | **Sample number^b^** |
| --- | --- | --- | --- | --- |
| 1 | FAL10255 | 1 | 36.5 | 1 |
| 1 | FAL10255 | 2 | 41.7 | 2 |
| 1 | FAL10255 | 3 | 33.1 | 3 |
| 1 | FAL10255 | 4 | 41 | 4 |
| 1 | FAL10255 | 5 | 28.6 | 5 |
| 1 | FAL10255 | 6 | 15.2 | 6 |
| 1 | FAL10255 | 7 | 35.4 | 7 |
| 1 | FAL10255 | 8 | 38.8 | 8 |
| 1 | FAL10255 | 9 | 26.9 | 9 |
| 1 | FAL10255 | 10 | 32.2 | 10 |
| 1 | FAL10255 | 11 | 27.5 | 11 |
| 1 | FAL10255 | 12 | 25.9 | 12 |
| 2 | FAL10255 | 13 | 25.9 | 12 |
| 2 | FAL10255 | 14 | 47.2 | 13 |
| 2 | FAL10255 | 15 | 54.4 | 14 |
| 2 | FAL10255 | 16 | 39.1 | 15 |
| 2 | FAL10255 | 17 | 37.2 | 16 |
| 2 | FAL10255 | 18 | 44.8 | 17 |
| 2 | FAL10255 | 19 | 45.8 | 18 |
| 2 | FAL10255 | 20 | 49 | 19 |
| 2 | FAL10255 | 21 | 50.2 | 20 |
| 2 | FAL10255 | 22 | 43.8 | 21 |
| 2 | FAL10255 | 23 | 52.7 | 22 |
| 2 | FAL10255 | 24 | 54.1 | 23 |
| 3 | FAL82590 | 1 | 47.5 | 24 |
| 3 | FAL82590 | 2 | 43.9 | 25 |
| 3 | FAL82590 | 3 | 42.4 | 26 |
| 3 | FAL82590 | 4 | 37.9 | 27 |
| 3 | FAL82590 | 5 | 40.7 | 28 |
| 3 | FAL82590 | 6 | 54.6 | 29 |
| 3 | FAL82590 | 7 | 46.9 | 30 |
| 3 | FAL82590 | 8 | 43.7 | 31 |
| 3 | FAL82590 | 9 | 47.6 | 32 |
| 3 | FAL82590 | 10 | 54.1 | 33 |
| 3 | FAL82590 | 11 | 30.5 | 34 |
| 3 | FAL82590 | 12 | 37.2 | 35 |
| 4 | FAL82590 | 13 | 40.8 | 36 |
| 4 | FAL82590 | 14 | 43.5 | 37 |
| 4 | FAL82590 | 15 | 41.7 | 38 |
| 4 | FAL82590 | 16 | 48.3 | 39 |
| 4 | FAL82590 | 17 | 49.9 | 40 |
| 4 | FAL82590 | 18 | 37.5 | 41 |
| 4 | FAL82590 | 19 | 29.4 | 42 |
| 4 | FAL82590 | 20 | 34.3 | 43 |
| 4 | FAL82590 | 21 | 42 | 44 |
| 4 | FAL82590 | 22 | 38.5 | 45 |
| 4 | FAL82590 | 23 | 51.3 | 46 |
| 4 | FAL82590 | 24 | 35.8 | 47 |

^a^The barcode numbers represent the barcodes which are provided by Oxford Nanopore Technologies in the native barcoding kits (EXP-NBD104, EXP-NBD114). ^b^Sample 12 was repeated in the second run due to insufficient barcode ligation for this sample in the first run.
