# Supplementary material for: Genotyping of familial Mediterranean fever gene (MEFV)—Single nucleotide polymorphism—Comparison of Nanopore with conventional Sanger sequencing
Source: PLoS One. 2022 Mar 17;17(3):e0265622. doi: 10.1371/journal.pone.0265622 (PMC8929590; doi:10.1371/journal.pone.0265622)
Supplement: Attachment — Submitted filename: reviewer_comments_1-19-22.pdf [file pone.0265622.s008.pdf]

General commentary:

Summary: This paper assesses the accuracy of SNP genotyping in the coding region of the *MEFV* gene using the hand-held MinION device. Amplicon sequencing data for patient samples produced via nanopore sequencing were compared to the gold-standard for SNP genotyping (i.e., Sanger sequencing). The resultant data and subsequent analyses provide a bridge between current clinical practices and the accurate detection of disease-associated SNPs throughout the coding region of the *MEFV* gene using the MinION device to ultimately increase throughput and decrease cost.

Major strengths: This manuscript is straightforward, well-structured and presents the data in a comprehensive manner. Moreover, the data analysis is simple in both approach and scope. If the automated pipeline developed herein is made accessible, this could be easily repeated in a clinical setting (by individuals that may not have extensive knowledge about nanopore data analysis).

Major weaknesses: My main concern is that despite the clinical applicability of the research at hand, the significance of the results obtained are dampened by lack of consistency in the methods utilized (e.g., DNA input for PCR, equimolar amplicon pooling). Given that this study is largely focused on concordance of Sanger and nanopore sequencing, it is critical that the authors confirm the SNP in question using the current gold-standard.

Line-by-line commentary

Line 59: "This sequencing approach offers the advantages of real-time sequencing, ultra-long read length (average read length up to 10 kb), high throughput and low material requirements..."

> *The authors mention low material requirement as an advantage of ONT sequencing but 7uL of each amplicon were used for Sanger (Line 116) and 10uL for nanopore (Line 127). This is counter to their claim here; can the authors please elaborate?*

Line 70: "This can be done either by testing for the most common mutations (targeted mutation analysis) or by sequencing of selected exons."

> *It would be helpful if the authors expanded upon the discussion surrounding current diagnostic techniques (e.g., method, number of SNPs assessed in a clinical setting) as well as the technological limitations in the introduction.*
> *Where does NGS (e.g., Illumina) fit in? Has it been used for the diagnosis of MEFV in a clinical setting?*

Line 74: "FMF is inherited autosomal recessive and results from point mutations (single substitutions) in the Mediterranean Fever (MEFV) gene."

> *Are all SNPs associated with FMF located within the exons of this gene? If not, please provide an explanation as to why the authors chose to focus exclusively on the exons (i.e., diagnostic value).*

Line 107: **"**PCR amplification of the MEFV target regions was performed stepwise in eight different PCR reactions..."

> *How much DNA was used per reaction? Were samples quantified before amplification? Why or why not? This information is critical to the repeatability and reproducibility of this work.*

Lines 115 & 130: Question- *Why were 2 different PCR purification methods used prior to Sanger (ExoSAP-IT) and nanopore sequencing (AMpure XP beads)?*

Line 134: **"**The libraries were prepared with an identical number of samples..."

> *How many samples per flow cell? How did you pick this number? What was the DNA input per sample for library prep and how much of this was pooled prior to sequencing? Again, this information is critical for repeatability and reproducibility.*

Line 161: **"**Once the automated data analysis pipeline was complete..."

> *Is the automated data analysis pipeline developed here available on github?*

Figure 2 & Table S1: Comment: *It would be beneficial to include amplicon length.*

Table 2: *11 sequencing reactions must be performed to sequence all target regions with Sanger sequencing.*

> *Why are 11 sequencing reactions required when only 8 amplicons were generated via PCR?*

Line 249: **"**...it was possible to sequence the relevant regions of all MEFV exons with a very high read depth."

> *What do the results suggest about read depth requirements for accurate SNP genotyping? It is important to mention that this could be achieved by increasing sample per flow cell which would also decrease price per sample (maybe a future direction). However, the authors should also provide the number of samples were multiplexed in the first place.*

Line 263: "Nevertheless, because a comprehensive data-base research did not reveal any information about this transversion and we could not confirm its presence by Sanger sequencing..."

> *While this is a valid explanation, supporting data is required. Would it be possible to confirm via Sanger sequencing using a different set of primers to produce an amplicon centered on the SNP in these two samples? This would significantly strengthen the results obtained and further highlight the advantages of nanopore sequencing.*

Line 295: Question: *What benefits do R10.4 flowcells offer the region of interest?*

Line 271: Comment: *A major flaw of this work is failure to harness the long-read capabilities of ONT sequencing platforms. Previous studies have demonstrated that amplification of the entire gene is in fact feasible. This would also bypass the need for multiple PCR reactions, reduce variability in amplicon coverage, and enabling phasing of the SNPs. Would this information provide valuable diagnostic and therapeutic value in a clinical setting? If so, these researchers should have used primers to generate overlapping amplicons that spanned the entire gene in PCR reactions containing the same amount of DNA at minimum. Because long reads are mentioned as an advantage of nanopore sequencing throughout, these points should be included as a limitation in the discussion section.*

The development of a standardized workflow for sample processing should also be mentioned as a requirement prior to clinical applicability to bypass these limitations.
